# The Clinical Concept of Opioid Addiction Since 1877: Still Wanting After All These Years

**DOI:** 10.3389/fpsyt.2018.00508

**Published:** 2018-10-16

**Authors:** Christian G. Schütz, Susana Ramírez-Vizcaya, Tom Froese

**Affiliations:** ^1^Department of Psychiatry, University of British Columbia, Vancouver, BC, Canada; ^2^Philosophy of Science Graduate Program, National Autonomous University of Mexico, Mexico City, Mexico; ^3^Institute for Applied Mathematics and Systems Research, National Autonomous University of Mexico, Mexico City, Mexico

**Keywords:** addiction, opioid use disorders, psychopathology, habits, 4E cognition, enactivism, anosognosia

## Abstract

In 1877, the psychiatrist Edward Levinstein authored the first monograph on opioid addiction. The prevalence of opioid addiction prior to his publication had risen in several countries including England, France and Germany. He was the first to call it an illness, but doubted that it was a mental illness because the impairment of volition appeared to be restricted to opioid use: it was not pervasive, since it did not extend to other aspects of the individuals' life. While there has been huge progress in understanding the underlying neurobiological mechanisms, there has been little progress in the clinical psychopathology of addiction and in understanding how it relates to these neurobiological mechanisms. A focus on cravings has limited the exploration of other important aspects such as anosognosia and addiction-related behaviors like smuggling opioids into treatment and supporting the continued provision of co-patients. These behaviors are usually considered secondary reactions, but in clinical practice they appear to be central to addiction, indicating that an improved understanding of the complexity of the disorder is needed. We propose to consider an approach that takes into account the embodied, situated, dynamic, and phenomenological aspects of mental processes. Addiction in this context can be conceptualized as a habit, understood as a distributed network of mental, behavioral, and social processes, which not only shapes the addict's perceptions and actions, but also has a tendency to self-maintain. Such an approach may help to develop and integrate psychopathological and neurobiological research and practice of addictions.

## Introduction

The last decades have seen a great deal of progress in our understanding of the underlying neurobiological mechanisms of opioid and other substance use disorders and on the perception of addiction as a public health issue. However, we believe that the clinical psychopathology of addiction has undergone scant development. In our view addiction is a mental disorder. However, many, including psychiatrists, often seem to struggle to support this statement. We contend that this resistance is attributable to an inadequate scientific theory of the psychopathology of addiction, and especially a restricted conception of the addicted mind as core problems in the current discussion of addiction, including opioid addiction.

One hundred and forty years ago, Edward Levinstein, Director of the *Maison de Santé* in Berlin, published a monograph entitled *Die Morphiumsucht* (1). This was the first monograph identifying opioid addiction as a disorder. One year later it was translated into English (2) and French (3). Around 1853, injectable morphine had become available in Germany, and 25 years later, Germany, like other countries, was experiencing a wave of problems with non-prescribed injection of opiates (4).

Levinstein's definition of addiction as an “uncontrollable desire to use” has held up in the last 140 years. He argued that morphine addiction is a disease, but not a mental disease, because the *will* of the addicted individual was not *pervasively* damaged. Individuals could use their will successfully in the context of work and family, but not with opiates (2). Conversely, current versions of the Diagnostic and Statistical Manual of Mental Disorders (DSM) and the International Classification of Diseases (ICD) include opioid use disorders as *mental* disorders. Nevertheless, a great deal of ambivalence remains toward the psychiatric diagnosis of substance use as a mental disorder, not only in the general population, but also among health professionals in general, including psychiatrists (5–8). In our view, this ambivalence reflects the lack of a comprehensive theory of addiction that takes into account the full complexity of the phenomenon in its neurobiological, psychological, and sociocultural aspects.

In this perspective paper, we undertake a critical appraisal of the current status of the psychopathology of opioid use disorder from a clinical point of view. We further suggest that recent developments in cognitive science, in particular enactivism (9, 10), serve as a suitable framework to overcome some of the shortcomings of the current approach by providing a more comprehensive model of addiction that integrates life and social sciences, dynamical and complex systems theory, and philosophical-phenomenological approaches.

## Limitations of the current psychopathology of addiction

Most physicians and psychiatrists would find it difficult to respond if asked about the nature of addiction. We propose that this difficulty can be attributed to a lack of a mature theory regarding the clinical psychopathology of addiction. Searches for articles using the keywords *psychopathology* and *addiction* or *substance use disorder* will mainly retrieve articles on psychopathology of other mental disorders and concurrent addiction, but not about the psychopathology of addiction as such. We think that a glance at the origin and development of psychopathology and the concept of mental disorders may help to understand this deficit.

The original development of the concepts of psychopathology and mental disorders has been attributed to Karl Jaspers. Jaspers based his concept of psychopathology on Edmund Husserl's phenomenology, focusing on conscious experiences and excluding non-conscious aspects (11). He felt uncomfortable with Sigmund Freud's speculations of the impact of non-conscious aspects of the mind on salience, motivation and decision-making (12). Jasper's philosophical anchoring in phenomenology as a disciplined investigation of conscious experience seems to have been lost (13, 14). Instead, current clinical psychopathology (signs and symptoms) continues to be based on the naïve common sense concepts of the mind derived from folk psychology, with all its limitations and scientifically unsustainable assumptions (15). But even Jaspers in his original formulations made no attempt to systematically probe the phenomenology of actual lived experience of addiction as reported by addicted individuals.

In a recent attempt to develop such a phenomenology of addiction, Owen Flanagan talked about shame and normative failures, not pathological craving (16). Patients appear to experience cravings as intuitive drives, not as uncontrollable or *foreign* urges. This is different from other disorders, such as obsessive-compulsive disorder (OCD), where urges can be experienced as intrusive, overwhelming and dysfunctional. It appears that individuals with an addiction do not tend to spontaneously report a feeling of a “loss of control.” If individuals suffering from addiction step back and evaluate their lives, they can articulate the negative impact of their substance use, but this is distinct from a feeling of losing control due to craving. While this is a clinical observation not unfamiliar to treatment providers, data and studies regarding this phenomenon are lacking and current psychopathology of addiction has remained silent to it. Although clinical experience, including clinical psychopathology, cannot replace scientific evidence, clinical psychopathology is important to understand the expression of the disorder in a patient's life and to relate the neurobiological mechanisms to the relevant aspects of this clinical disorder.

*Sense of control* and *will* are central concepts in commonsense psychology, but they are surprisingly poorly conceptualized or investigated in current psychopathology. If pressed, professionals will express contradictory views: they will argue either that the will of an addict is in principle intact, as conceptualized by Levinstein (2); or that it is impaired (lack of willpower), as argued by Jaspers, Kraepelin and others (17, 18). This contradiction might arise because commonsense psychology endows an individual with a consistent, single will that is either “healthy” or “sick” in addiction. Imposing a dichotomy does not do justice to the complexity of agency. Discussion of the role of volition, will and agency is closely related to questions around free will. Free will is a conceptual cornerstone of the prevalent Western folk intuitions of individuals as responsible human beings (19–21). This is a deeply engrained perspective, and also may be a reason why addiction is associated with such a high level of stigma. The image of a hijacked brain, endorsed by the National Institute on Drug Abuse (NIDA) as a metaphor aimed at lay audiences, circumvents this stigma by describing the brain as seized by an unnamed outside agent (e.g., drugs or addiction processes) that forces it to follow a new trajectory.

It is undeniable that the brain undergoes neuroplastic changes in response to substance abuse. However, neuroplasticity does not imply that the brain has been “hijacked” (22). Furthermore, this metaphor may undermine the patients' possibility of taking at least partial responsibility for their actions and does little to support their capacity for change (23). Accepting to be completely controlled by drugs might contribute to a low self-efficacy in addiction. We think that this last point is relevant for recovery, since self-efficacy (24) has been found to be a significant determinant of behavior change and relapse prevention in studies on smoking cessation and alcoholism treatments (25–27). Pickard's framework of “responsibility without blame” (28), which proposes to change attitudes toward addiction by decoupling responsibility from morality, might be useful in clinical practice for avoiding stigma and blame without removing the patients' sense of agency. Additionally, a more nuanced theory of agency, which can defend the sense of being an autonomous individual, while acknowledging the constraints of biological embodiment also appears to be advantageous or even necessary, as it might help to identify healthy aspects of agency supporting a restructuring of an individual's life. In the next section, we will argue that enactivism can provide such a theory.

Another feature of addiction, which we feel needs more attention, is that it involves a host of characteristic behaviors beyond use itself. Levinstein already provided a broad range of examples of the effect of morphine addiction on the patients' responses and behavior, e.g., when the treatment provider has to expect that, independently of the “respectability” of the patients, they will try to smuggle morphine into treatment. He also pointed out that “hardly any person suffering from morbid craving for morphia^1^ is able truthfully to state the daily quantity of morphia used, and the hour when he last injected morphia” (2). Furthermore it seems to require considerable effort to switch from supporting substance use of others to supporting recovery and abstinence of others, even in the context of a joint recovery. These behaviors and social phenomena are familiar to anybody treating patients suffering from substance use disorders, and yet remain rarely discussed as an integrated part of the disorder. We believe that they once again point toward the need for a more elaborate and far-reaching theory of addiction.

Neuroscience will play an essential role in developing a more comprehensive conceptualization of addiction. For instance, some of the aforementioned aspects have been subsumed under the description of “denial” (29). Denial can be considered a refusal to accept reality or facts, acting as if an uncomfortable event, thought, or feeling does not exist (30). Recently, Nora Volkow and other authors (31–33) have touched upon denial in addiction by discussing *anosognosia*, conceptualizing it as a “dysfunction of brain networks subserving insight and self-awareness” (31). Another example is the theory of *allostasis*, developed by Sterling and Eyer (34) to explain the relationship between stress and diseases. George Koob and other researchers (35–38) have incorporated it into the field of addiction to explain the neurobiological mechanisms underlying vulnerability to drug addiction and relapse. According to this theory, a pathological equilibrium related to sustained changes in the stress response system or *allostatic load* (39) leads to a self-reinforcing drug use pattern. This theory explains compulsion and relapse as behaviors aimed at reestablishing hedonic homeostasis by relieving the allostatic load, which manifests itself as a spiraling affective tension resulting from withdrawal, repeated failures in self-regulation, and other daily stressors. Similar to the anosognosia concept, this framework captures aspects of addiction familiar to clinicians, but currently not covered by clinical psychopathology. One key feature about this theory is that it emphasizes the integral causality between the whole body and the environment, making it clear that the brain does not work in isolation, but only as a part of a complex system. This fact has also been acknowledged by Thomas Fuchs, who regards the brain as “an organ of mediation” between the organism as a whole and its environment (23).

In general, it can be said that acknowledgement of neurobiological aspects has had a very limited impact in the psychopathology of addiction. This may be partly because clinical psychopathology appears to be increasingly disconnected from biological psychiatry. In fact, from the perspective of the latter, psychopathology is sometimes considered a barrier for progress (40, 41). Psychiatrists conducting genomic and neuroscientific research have tried to circumvent it, creating new biological concepts such as endophenotypes (42) and the Research Domain Criteria (RDoC) (41). Behavioral neuroscience certainly is an essential source of progress for research on and treatment of addictions, but it does not replace clinical psychopathology. We see a need for both neuroscience and clinical psychopathology to more effectively inform each other to obtain a more comprehensive understanding of opioid use disorders and other addictions. In the forthcoming section, we suggest that one promising avenue for collaboration might come from an enactive approach to cognitive sciences.

## Toward a new understanding of opioid and other drug addictions

Recent developments in embodied, embedded, extended, and enactive (4E) cognitive science have done much to highlight how embodied interactions, tool-use, affectivity, language, material environment, and socio-cultural practices shape lived experience and the functioning of the mind. A theory of addictions based on 4E theory seems to be an attractive option to move the field forward.

Walter (43) recently described the 4E approach to cognition as the potential base for a third wave in biological psychiatry. By treating the mind/brain as embodied, embedded, extended, and enactive, processes external to the brain are considered to be constitutive of mental processes and thus also constitutive of disordered and pathological mental processes. We agree and see much promise of applying these insights to addiction. In particular, we propose that an *enactive* approach may do the clinical phenomena of addiction more justice, while also being consistent with biological findings.

Enactivism emerged as an alternative to current mainstream cognitive science, emphasizing the dynamical, self-organized, embodied, affective, intersubjective, and situated nature of cognition, as well as its phenomenological dimension (10). The enactive approach emphasizes the centrality of agency for understanding mind and behavior. An agent is understood as “a self-constructed unity that engages the world by actively regulating its exchanges with it for adaptive purposes that are meant to serve its continued viability” (44). This means that agents generate an *identity* through their activity in the world, and strive to preserve it in the face of external perturbations and in spite of its intrinsic precariousness and entropic trends. In order to do that, agents need to be *adaptive*, i.e., they need to regulate themselves to stay within the limits of their viability (44).

On a biological level, agents seek to preserve a metabolic identity in order to survive. However, in the case of humans, they also strive to maintain habitual identities (45). In this regard, according to enactivism, the preservation of habits constitutes a central source of normativity that guides an agent's perception, thought and behavior: agents will tend to avoid situations and actions that may threaten their habitual identities and to look for favorable ones (46). Accordingly, agents create meaningful relations with the world in the sense that everything that contributes to the conservation of their biological and habitual identity is seen as intrinsically *good* and *attractive*, while everything that challenges its subsistence as intrinsically *bad* and *aversive* (47).

This framework also suggests “bundles of habits” (48) constituting a complex network of *regional identities* that involve bodily and neural processes, as well as interactions with the material, social, and cultural environment. These identities mutually enable and restrain each other (49), giving rise through their interaction to a global identity i.e., a loosely assembled *self*.

Under this perspective, addiction is considered one of the many habitual identities that constitute an addict's form of life and that is so deeply ingrained into the agent's physiology that it alters her metabolic autonomy and escalates to dependence. In this sense, addiction can be regarded as a *bad or pathological* habit because it endangers or constrains some of the addict's other identities, such as the biological or social ones. In dynamical systems terms, it can be said that addicts are stuck in a suboptimal attractor, which creates a tension that may manifest as frustration or anxiety for not being able to develop other regional identities. This view thus places addiction within the self, and not as a compulsion or an alien force. Additionally, it acknowledges addicts as autonomous agents that strive to preserve an identity that they have forged through a long history of interactions with their material, social, and cultural environment.

This perspective helps to explain the puzzling but common behaviors of individuals initiating treatment, but smuggling drugs into it and failing to disclose the full extent of usage: these behaviors can be seen as ways of maintaining the addict's form of life, which is being threatened by treatment. Furthermore, addictions may be so difficult to override not only because of their self-sustaining character, but also because their dynamics influence the formation and maintenance of other related habits, including social ones, thus making it necessary to change many other regional identities and, eventually to perform more extensive reshaping of the addict's entire self and its narratives. In order to do this, the enactive approach emphasizes the need to take into account the embodied, affective, situated, intersubjective, and extended aspects of addiction, as well as its phenomenological and dynamical dimensions to achieve a broader understanding and an impact on treatment. We propose that these factors should be a prominent focus of future research on addiction.

While we argue for this approach within the context of a very “underdeveloped” clinical psychopathology, its value will only be realized if it can better integrate diverse aspects of the disorder, including psychopathology and neurobiological findings; if it can predict patients' trajectories; and if it facilitates the development of new effective treatments. One future line of research can come from relating this enactive perspective with the theory of allostasis. In this regard, for example, the enactive notion of *adaptivity*, understood as “the capacity of an organism to regulate itself with respect to the boundaries of its own viability” (44) can be conceptually linked to that of allostasis, which refers to the principle that “to maintain stability an organism must *vary* all the parameters of its internal milieu and match them appropriately to environmental demands.” (34). Additionally, both frameworks regard the brain as an interacting dynamical system embedded within larger ecological systems. In fact, the notion of allostasis has started to be incorporated within the enactive approach in relation to autonomy and self-regulation (50). We believe that this exchange will be mutually beneficial, for it can provide enactivism with a more solid physiological and empirical grounding and connect allostasis theory with science informed cutting-edge philosophy of mind.

## Conclusions

Levinstein's monograph ends with case histories. The last case is about Darius, who dies during treatment. The author suggests that he died because he relapsed and overdosed (2). This may be taken as a reminder of the high human and societal cost induced by addiction. A more mature theory of the “pathologies” of the mind, as well as their relationship to individuals' experiences, actions, and brain mechanisms seems to be urgently required. This need may be most pronounced in the field of substance use disorders, and it appears to be time to move beyond the traditional framework of “folk psychology” and brain mechanisms. The need to incorporate science-based and philosophically informed developments in understanding the mind, such as those suggested by the 4E approaches to cognition, appears to be more than a mere academic exercise; it might actually be considered a necessary step to successfully integrate and further develop preclinical neuroscience and clinical psychopathology.

## Author contributions

CS wrote the initial draft of the manuscript. SR-V expanded the section on new approaches to cognitive science. All authors worked on shaping the manuscript into its final form.

### Conflict of interest statement

The authors declare that the research was conducted in the absence of any commercial or financial relationships that could be construed as a potential conflict of interest.

## Footnotes

^1^The term “morphia” used in the English edition is equivalent to the term “morphine” that is employed nowadays.
